# Capability of polygonum cuspidatum extract in inhibiting AGEs and preventing diabetes

**DOI:** 10.1002/fsn3.1029

**Published:** 2019-05-01

**Authors:** Zhanwu Sheng, Binling Ai, Lili Zheng, Xiaoyan Zheng, Yang Yang, Yixiao Shen

**Affiliations:** ^1^ Haikou Experimental Station Chinese Academy of Tropical Agricultural Sciences Haikou China; ^2^ School of Nutrition and Food Science Louisiana State University Agricultural Center Baton Rouge Louisiana

**Keywords:** advanced glycation end products (AGEs), diabetes, Nε‐(Carboxymethyl)‐L‐lysine (CML), polygonum cuspidatum, protein glycation

## Abstract

Diabetes is a metabolic disorder disease associated with advanced glycation end products (AGEs) and protein glycation. The effect of polygonum cuspidatum extract (PE) on AGEs and Nε‐(Carboxymethyl)‐L‐lysine formation, protein glycation, and diabetes was investigated. Six primary phenolics in a range of 12.36 mg/g for ellagic acid to 0.01 mg/g for piceid were determined in PE. In an intermediate‐moisture‐foods model, inhibition rate of PE was as high as 54.2% for AGEs and 78.9% for CML under aw 0.75. The protein glycation was also inhibited by PE. In a diabetic rat model, the levels of blood glucose, serum malondialdehyde, cholesterol, triglycerides, and low‐density lipoproteins were effectively reduced by PE treatment. The antioxidation capacity (T‐AOC) and superoxide dismutase (SOD) activity were also mediated by PE. Additionally, the activates of liver function‐related enzymes including alkaline phosphatase (ALP), glutamate pyruvate transaminase (GPT), and glutamate oxaloacetate transaminase (GOT) in diabetic rat were improved by PE.

## 1 INTRODUCTION

Polygonum cuspidatum is an herbaceous perennial plant in the buckwheat and knotweed family Polygonaceae grown in Asia and North America (Zhang, Li, Kwok, Zhang, & Chan, 2013). It is commonly used as food seasonings with a slight sour taste in China and Japan (Peng, Qin, Li, & Zhou, 2013). Abundant in phytochemicals and some bioactive compounds, it has been reported to generate antioxidant activities (Kuo et al., 2013). Also, polygonum cuspidatum aqueous extracts exhibited potent estrogenic activity and antibacterial activity (Lazurca, Lazurca, Fetea, Ranga, & Socaciu, 2012). The ethanolic extract of polygonum cuspidatum shows inhibitory effect on hepatitis B virus in a HBV‐producing cell line (Chang et al., 2005). From the therapeutic perspective, polygonum cuspidatum roots and rhizomes have been used for the suppressing cough, treating hepatitis, jaundice, arthralgia, and hyperlipidemia scald, as well as to promote blood circulation (Zhang et al., 2013).

Glycation (nonenzymatic glycosylation) is a binding reaction between the carbonyl group of a reducing sugar and an amino group of proteins, lipids, or peptides and finally generates the heterogeneous compounds advanced glycation end products (AGEs) (Masaki, Okano, & Sakurai, 1999). AGEs exert adverse effects on cell functions based on the mechanisms of free radicals production, protein or lipid fragmentation, enzyme activity alternation, and immunogenicity modification (Khangholi, Majid, Berwary, Ahmad, & Aziz, 2016). Thus, it contributes to the development and progression of diabetes complications, including atherosclerosis, retinopathy, nephropathy, and neuropathy (Khangholi et al., 2016). Diabetes is also caused by insufficient insulin secretion due to dysfunction of pancreas which was reflected by the increasing level of blood glucose, hyperlipidemia, and liver function impairment (Maria, Campolo, & Lacombe, 2015). Therefore, inhibition of AGE formation and control of blood glucose, lipid parameters, and key liver enzyme are considered as the therapeutic approaches for diabetic patients. Traditional diabetes treatments require pharmacological agents which cause side effects such as gastrointestinal issues, weight gain, hypoglycemia, and syndrome of inappropriate antidiuretic hormone (Mitri & Hamdy, 2009). Thus, medicinal plants have been investigated for remedy purpose on different biological system disorders. However, a comprehensive study on the phytochemicals and anti‐AGE formation and antidiabetes potential of polygonum has not been well documented. Therefore, the primary phenolics of polygonum and the potential of its extracts in AGE inhibition, and diabetes treatment were investigated in this study. It will provide important information for further application of herbal polygonum as a natural therapy for pharmacological and drug development purpose.

## 2 MATERIALS AND METHODS

### 2.1 Chemicals and materials

p‐Coumaric acid, ellagic acid, piceid, coumarin, emodin and cinnamic acid standards, sodium chloride, sodium bromide, glucose, glycerol, streptozotocin (STZ), sodium azide, thionyl chloride, dichloromethane, and trifluoroacetic acid anhydride were purchased from Sigma‐Aldrich. HPLC grade methanol, ethanol, acetic acid, acetonitrile, chloroform, acetone, as well as hydrochloric acid were ordered from Thermo Fisher Scientific Co. Whey protein isolate (WPI) was purchased from Davisco Foods International, Inc. (Eden Prairie, MN). Nε‐(Carboxymethyl)‐L‐lysine (CML) standard was supplied by Toronto Research Chemicals Inc.

### 2.2 Preparation of the aqueous polygonum extract

Fresh polygonum leaves were harvested from a local farm (Haikou, China) and ground in liquid nitrogen. The aqueous extract was obtained by extracting the ground polygonum (300 g) with 400 ml ethanol (70%) using Soxhlet apparatus for 24 hr as described in the study of Bokaeian, Nakhaee, Moodi, Farhangi, and Akbarzadeh (2010). The extract solution was then filtered and evaporated by a vacuum centrifuge evaporator (Labconco). The dried polygonum extract was stored at −20°C until use.

### 2.3 Determination of major phenolics in polygonum extract

An aliquot of polygonum extract was redissolved in methanol to make a working solution with concentration of 2 mg/ml. It was determined using a high‐performance liquid chromatography (HPLC) system (Waters 2690) coupled with a C18 column (id 250 × 4.60 mm 5 micron; Phenomenex) and a photodiode array detector. The HPLC operation condition was based on the study of Shen, Prinyawiwatkul, Lotrakul, and Xu (2015). The major phenolic compounds were identified and quantified based on the retention times, spectra, and calibration curves of their corresponding commercial standards.

### 2.4 Determination of AGEs, CML, and protein glycation

#### 2.4.1 Preparation of Protein−Sugar‐Rich intermediate‐moisture‐foods (IMFs)

For the control group, the IMFs (200 g) consisted of 90 g of WPI, 60 g of glycerol, 25 g of glucose, and 25 g of DI water and then mixed homogeneously. Sodium azide (80 mg) was added in the IMF dough to prevent microbial growth. For the treatment group, polygonum cuspidatum extract was fortified at a concentration of 10 mg/g (PE1), 20 mg/g (PE2), or 40 mg/g (PE3) to the IMF dough and was followed by the addition of same amount of glucose, water, and glycerol as control group. It was homogenized through vigorous stirring before the same amount of WPI and sodium azide were added. Both control and fortified IMF dough were placed on a Petri dish separately and laid on a rack in an airtight plastic box. The experiment was carried out under different water activity (aw) conditions (aw 0.75 and aw 0.56) which were prepared by saturated sodium chloride solution and sodium bromide solution, respectively. All of the boxes were sealed and incubated at 45°C. Ten grams of each dough was collected for analysis at days 0, 7, 14, 21, 28, and 45 for AGEs; days 0, 7, 14, 21, and 45 for CML; and days 1, 3, 5, and 7 for protein glycation.

#### 2.4.2 Determination of AGEs

The control IMF or fortified IMF sample (500 mg) was extracted by dissolving in 10 ml of double distilled water (DDW). After magnetic stirring at room temperature for 80 min, the extract solution was centrifuged at 4,000 *g* for 30 min. Four milliliters of the supernatant was collected for AGE determination. The fluorescence intensity was measured by an F‐4500 Luminescence Spectrometer (Shimadzu, Japan) at the excitation wavelength of 370 nm and emission wavelength of 440 nm with a slit width of 5 nm. Level of AGEs in the sample was expressed by the fluorescence intensity.

#### 2.4.3 Determination of CML

The level of CML was determined by GC‐MS method as described in the study of Sheng et al. (2016) with minor modification. Control IMF or fortified IMF sample (200 g) was defatted with 20 ml of chloroform/acetone solvent (1:3, v:v). After vortexed and sonicated for 10 min, the mixture was centrifuged at 4,000 *g* for 15 min. The precipitated protein was collected and dried. Then, it was hydrolyzed by hydrochloric acid solution (8 ml of 6 mol/L) at 110°C for 24 hr. Fifty microliters of protein hydrolysate was dissolved in 1.0 ml of DDW, filtered, and dried. The extract was reacted with 1 ml of thionyl chloride/methanol (v: v, 1.46:100) at 110°C for 30 min and dried again. Derivatization was performed by adding 2 ml of dichloromethane and 400 μl of trifluoroacetic acid anhydride to the dried extract and incubated at room temperature for 1 hr. The CML in the extracted sample was determined by GC‐MS (Agilent 7890B, 7693 mass selective detector single quadrupole mass spectrometer system) coupled with an HP‐5MS column (30 m × 0.25 mm × 0.25 μm, Palo Alto, CA). The GC program was described as follows: initial oven temperature was set at 40°C and held for 1 min. Then, it increased to 70°C at the rate of 20°C/min, ramped to 300°C at the rate of 50°C/min, and held for 2 min. High purity helium was used as carrier gas with a flow rate set at 1.20 ml/ min. The transfer line temperature and ion source temperature were set at 250 and 230°C, respectively. The MS was operated in electron ionization (EI) mode with electron energy 70 eV and ion scan range of m/z 40−800. A calibration curve of CML was used for quantification.

#### 2.4.4 Determination of protein glycation

The degree of glycated protein was determined by LC‐MS (Waters UPLC ZMD 4000 (Waters Co.) and TOF mass spectrometer). Control IMF or fortified IMF (300 g) was extracted by dissolving in 15 ml of DDW and stirred at room temperature for 80 min. After centrifugation at 4,000 *g* for 30 min, 100 µl of the supernatant was collected and diluted 10 times with DDW then filtered. The BEH C18 column (2.1 × 100 mm, Waters Co.), mobile phase A (100% acetonitrile) and B (formic acid, 0.1%, v/v) with the flow rate of 0.3 ml/min were used. The gradient program was described as B% decreased from 80% to 50% from 0 to 15 min; 50% to 0% from 15 to 20 min; 0% to 80% from 20 to 21 min; and equilibrated at 80% from 21 to 23 min. The electrospray ion source was in positive mode at a spray voltage at 4.1 kV.

### 2.5 Animals experiment and blood sample analysis

Healthy male Sprague–Dawley (origin) rats with the weight ranging from 180 to 200 g were used in the animal experiment. A total of 24 rats were randomly divided into the following three groups of 8 animals each: group I, normal control (NC); group II, diabetic control (DC); group III, PE treatment. Animals were maintained in environmentally controlled conditions with a 12/12‐hr light/dark cycle at temperature of 23°C and relative humidity of 55 ± 5%. All the rats were housed in cage with free access to food and water. After 1 week's adaptive feeding with the basic diet, the rats were fasted for 12 hr and received intraperitoneal injection of streptozotocin (STZ) at a dose of 45 mg/kg body weight except the rats in the NC group. At 72 hr after injection, the fasting blood glucose level higher than 16.7 mmol/L was considered as DC group. NC and DC continued with basal diet consist of 7% of fat, 13% of protein, and a highly digestible starch for 4 weeks. Oral gavage of PE was performed in treatment group at the level of 0.2 g/ kg of body weight once a day for 4 consecutive weeks.

Blood was collected from tail vein for determination of glucose level every week during the experimental period. After 4 weeks, all of the rats were sacrificed and blood samples were drawn through heart puncture. The blood sample was then centrifuged at 1,500 *g* for 10 min, and the supernatant serum was collected for further analysis. Levels of triglycerides (TG), cholesterol (CHO), low‐density lipoprotein cholesterol (LDL‐C), high‐density lipoprotein cholesterol (HDL‐C), and activities of liver enzymes alkaline phosphatase (ALP), glutamic oxaloacetic transaminase (GOT) and glutamic pyruvic transaminase (GPT), as well as antioxidant enzyme superoxide dismutase (SOD), antioxidation capacity (AOC), malonaldehyde (MDA) were determined by commercial kits. Experimental procedures were approved and complied with the Chinese Code of Practice for the Care and Use of Animals for Scientific Purposes.

### 2.6 Data analysis

Value was expressed as mean ± standard deviation with triplicates for each determination. The experimental data were analyzed using ANOVA (General Linear Model procedure, SAS system, SAS 9.1.3). The significant differences among treatments were conducted at *p* < 0.05 (SAS, 9.1.3).

## 3 RESULTS AND DISCUSSION

### 3.1 Primary phenolics in polygonum cuspidatum extract

**Figure 1:**
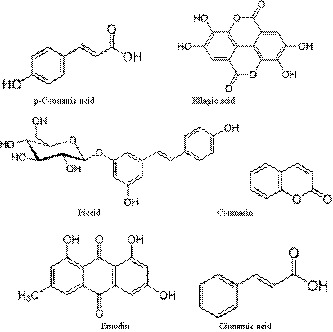
Chemical structures of major phenolic compounds in polygonum cuspidatum extract

**Figure 2:**
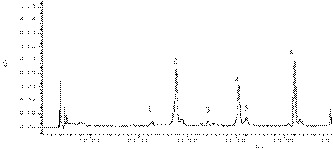
Chromatogram of polygonum cuspidatum extract under the wavelength of 280 nm: 1. p‐coumaric acid (25.6 min); 2. ellagic acid (27.7 min); 3. piceid (34 min); 4. coumarin (41 min); 5. emodin (41.5 min); 6. cinnamic acid (52 min)

In this study, a total of six primary phenolics including *p*‐coumaric acid, ellagic acid, piceid acid, coumarin, salicylic acid, and cinnamic acid were identified in polygonum extracts. The chemical structures and HPLC chromatograms of the phenolic compounds are shown in Figure 1 and Figure 2. Ellagic acid and cinnamic acid were the highest two phenolics with the concentration of 12.36 ± 0.93 mg/g DW and 12.05 ± 0.67 mg/g DW, respectively, which was followed by coumarin (8.40 ± 0.41 mg/g DW). The concentrations of *p*‐coumaric, emodin, and piceid were in a descending order of 2.56 ± 0.03, 1.17 ± 0.01, and 0.74 ± 0.01 mg/g DW, respectively (Table 1). It has been reported that ellagic acid plays cardioprotective, hepatoprotective, and gastroprotective roles in human body (Beserra et al., 2011). Meanwhile, *p*‐coumaric acid has been proved to generate antiaging, anticancer, and antidiabetes functions (Saibabu, Fatima, Khan, & Hameed, 2015). Piceid, a precursor of resveratrol, had anticarcinogenic effects, antioxidation activity, and inhibition of platelet aggregation (Su et al., 2013). Additionally, coumarin has anti‐inflammatory, antiviral, and antihypertension effects (Venugopala, Rashmi, & Odhav, 2013). Moreover, cinnamic acid is associated with a beneficial influence on diabetes and its complications as well as antioxidant ability (Adisakwattana, 2017). Belonging to the same *Polygonaceae* family, Rheum had *p*‐coumaric acid of 9–26 µg/g DW and buckwheat had cinnamic acid of 3.10–3.70 mg/g DW, which were significantly lower than those in polygonum, respectively (Wiczkowski et al., 2016). Compared with other herb plants, polygonum cuspidatum had higher concentration of coumarin (8.40 ± 0.41 mg/g DW) than guaco (0.775–1.131 mg/g DW) and higher ellagic acid (12.36 ± 0.93 mg/g DW) than geum (0.44–0.57 mg/g DW) (de Melo & Sawaya, 2015; Owczarek, Olszewska, & Gudej, 2015).

**Table 1:** Major phenolic compounds in the polygonum cuspidatum extracts

Peak No.	Compounds	Concentration (mg/g DW)
1	*p*‐Coumaric acid	2.56 ± 0.03c
2	Ellagic acid	12.36 ± 0.93e
3	Piceid	0.74 ± 0.01a
4	Coumarin	8.40 ± 0.41d
5	Emodin	1.17 ± 0.01b
6	Cinnamic acid	12.05 ± 0.67e

Abbreviation: DW, Dry weight basis.

Different letters in the table indicate a significant differences (*p* < 0.05).

### 3.2 Effect of polygonum cuspidatum extract on AGEs, CML, and glycated protein formation

**Figure 3:**
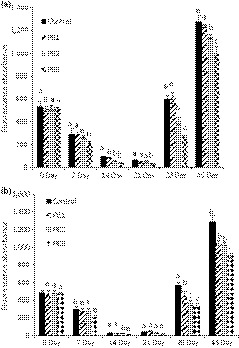
Fluorescence absorbances of AGEs in control and polygonum cuspidatum extract treatments under (a) aw 0.75 and (b) aw 0.56

**Figure 4:**
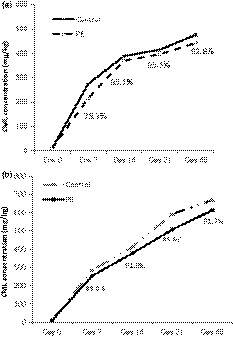
Concentrations of CML in control and PE treatment during storage under aw (a) 0.75 and (b) 0.56

The advanced glycation end products (AGEs) are mainly generated from glycation, with the reaction of free amino group of protein and carbonyl group of reducing sugar. A typical AGE compound CML can be generated from either reaction of Amadori products and amino acids or oxidative breakdown of α‐oxoaldehydes mediated polyol pathway (Goldin, Beckman, Schmidt, & Creager, 2006). Thus, total AGEs and CML have been recognized as reliable biomarkers of oxidative damage and pathogenetic factors of some oxidative‐based diseases such as diabetes, atherosclerosis, and Alzheimer's disease (AD), since a positive correlation between the accumulation of AGEs in tissues/fluids and those disease was observed (Younessi & Yoonessi, 2011). The AGE level in control and all treatments showed a decreasing trend in the first 14 day under both aw (Figure 3a,b). It was due to some glycosylation products from early storage of high protein food which had no fluorescence absorbance (Poulsen et al., 2013). During the early stage of Maillard reaction, the consumption of some amino acids also caused the decrement of fluorescence intensity (Poulsen et al., 2013). However, when incubation time reached 28 day, AGEs significantly increased under both aw (Figure 3a,b). It was because more reactive compounds were generated and reacted with free amino groups and thiol groups of protein. It dramatically increased AGE levels (Wu, Hsieh, Wang, & Chen, 2009). Figure 3a shows polygonum cuspidatum extract was the most effective at 28 day in inhibiting AGEs. It reduced by 32.0% and 54.2% in PE2 and PE3 treatments, respectively, under aw 0.75. Similarly, PE2 and PE3 reduced 34.6% and 39.7% of AGEs at 28 day, respectively, under aw 0.56 (Figure 3b). In the CML assay, the polygonum extract (PE3) had the highest inhibition rate at 7 day, which was 78.9% under aw 0.75 (Figure 4a). However, the CML was only slightly inhibited under aw 0.56 at 7 day and there was no significant difference at different times (Figure 4b). AGEs at the early glycation stage are known to associate with increased superoxide radical and hydroxyl radical production, especially with the presence of transition metals (Suantawee et al., 2015). Antioxidants are able to inhibit the radical‐based reactions involved in AGE formation and quench radicals which act as AGE precursors (Aldini et al., 2013). Thus, the phenolic compounds such as ellagic acid, coumarin, and coumaric acid in polygonum cuspidatum extract contributed to the inhibition of AGEs by scavenging free radicals (Priyadarsini, Khopde, Kumar, & Mohan, 2002; Teixeira, Gaspar, Garrido, Garrido, & Borges, 2013). It was in agreement with the result in the study of Liu et al. (2014) that the ellagic acid in pomegranate fruit extract contributed to the anti‐AGE effect.

**Figure 5:**
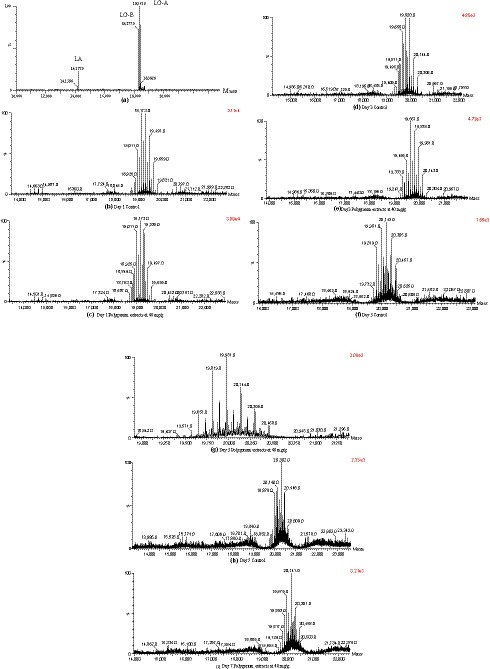
Mass spectrum of protein glycation in control and PE treatment during storage

Nonenzymatic glycation reaction proceeds slowly through different stages. Thus, during the reaction, the radicals also participate in accelerating the protein glycation by oxidizing side chains of amino acid residues of protein (Suantawee et al., 2015). It further leads to the formation of protein‐bound carbonyl or the loss of protein thiol group which causes protein oxidative damage (Elosta, Slevin, Rahman, & Ahmed, 2017). As a result, free radicals scavenging ability would be important for antiglycation purpose, especially at the early stages of glycation. In this study, the influence of polygonum cuspidatum extracts against fructose‐medicated nonenzymatic glycation was investigated based on the IMF model and only PE3 treatment. The protein glycation degree was evaluated by the modification of primary components α‐lactalbumin (LA), α‐lactoglobulin (LG‐A), and β‐lactoglobulin (LG‐B) in WPI protein during storage at 1, 3, 5, 7, and 21 day under aw 0.75. The molecular weights of LA, LG‐A, and LG‐B were 14,177, 18,362, and 18,277 Da, respectively (Figure 5a). At day 1, there was no significant difference between control and PE3 since the most abundant glycated proteins in these two groups were LG‐B+5G (19,173 Da) and LG‐B+4G (19,011 Da) which were 5 and 4 glucose bound with LG‐B, respectively (Figure 5b,c). At day 3, LG‐A+8G (19,820 Da) showed the highest intensity in control, which had one more glucose molecule bound to the LG‐A compared with LG‐A + 7G (19,657 Da) in PE3 treatment (Figure 5d,e). At day 5, higher molecular weight glycated LG‐A, LG‐A+10G (20,143 Da) was observed in control than LG‐A+9G (19,981 Da) in PE3 treatment (Figure 5f,g). Similarly, the highest intensity of glycated protein was LG‐A+11G (20,302 Da) in control, while it was LG‐A+10G (20,141 Da) in PE3 treatment (Figure 5h,i). The antiglycation ability of polygonum cuspidatum extract might be attributed to its abundant phenolics. The free radical scavenging activities played as the antioxidant role in the chain‐breaking performance on glycoxidation as exemplified in the study of Ardestani and Yazdanparast (2007) and Suantawee et al. (2015). For example, ellagic acid has been reported to inhibit AGE formation in diabetic mice and decrease the level of CML (Raghu, Akileshwari, Reddy, & Reddy, 2017). Coumarin has also been demonstrated to have antiglycation effect because the hydroxyl group of aromatic ring in coumarin molecule hinders the incorporation of albumin with glucose, thereby interferes with glycation and AGE formation (Aminjafari et al., 2016). In the study of Adisakwattana, Sompong, Meeprom, Ngamukote, and Yibchok‐anun (2012), approximately 35% of AGEs was inhibited by cinnamic acid in the incubation of BSA and fructose. Additionally, protein glycation was attenuated by cinnamic acid via reducing oxidation of thiol group oxidation and suppressing protein carbonyl formation (Adisakwattana et al., 2012). It has been evidenced that individual phenolic compounds could act synergistically in the crude extract of polygonum (Amin et al., 2016). Several edible plants including red grape skin, pomelo, and Cyperus rotundus have been proved to prevent reducing sugar‐mediated protein glycation (Ardestani & Yazdanparast, 2007; Caengprasath, Ngamukote, Makynen, & Adisakwattana, 2013; Jariyapamornkoon, Yibchok‐anun, & Adisakwattana, 2013). Due to the abundant phenolic and flavonoids such as catechin, epicatechin, quercetin, rutin, kaempferol, ellagic acid, gallic acid, and p‐coumaric acid, the consistent inhibition of protein glycation was observed in both in vitro and in vivo models (Atawodi et al., 2011). Also, the stilbene glucoside compounds in *Polygonum multiforum Thun* has been reported to effectively trap MGO and inhibit protein glycation (Ho & Wang, 2013).

### 3.3 Effect of polygonum cuspidatum extract on oxidative stress, blood glucose, lipid profile, and liver function of diabetic rats

#### 3.3.1 Oxidative stress

Oxidative stress is caused by an imbalance between excessive reactive species such as reactive oxygen species (ROS) and the natural antioxidant defense in biological system because of tissue damage or cell death (Rajeshwari, Shobha, & Andallu, 2013). The production of ROS is usually in balance with the availability antioxidant enzymes (Birben, Sahiner, Sackesen, Erzurum, & Kalayci, 2012). SOD is the main protective enzyme in the antioxidation system, which can catalyze the disproportion reaction of the superoxide anion and prevent damages to tissues caused by the superoxide anion (Ding, Wang, Song, & Zhou, 2017). Besides, malondialdehyde (MDA) is one of the end products with low‐molecular‐weight, so the increased MDA level can be also an indicator of oxidative stress (Ding et al., 2017). Thus, oxidative stress could determine the onset and progress of late diabetes complications (Rajeshwari et al., 2013). In this study, under the oxidative stress induced by STZ, the abnormal level of MDA was observed (Table 2). The DC had significant higher level of serum MDA (8.43 ± 0.53 nmol/L) than NC (6.74 ± 0.49 nmol/L); however, it was 6.05 ± 0.24 nmol/L in PE which was back to normal level (Table 2). The SOD activity was also slightly reduced in PE (0.48 ± 0.05 U/mL) compared with DC (0.54 ± 0.04 U/mL) (Table 2). Generally, the augmentation and propagation of oxidative stress lead to a simultaneous increase in free radical production (Adisakwattana et al., 2012). Thus, the great performance of polygonum cuspidatum extract was due to multiple phenolic compounds which played antioxidant or free radical scavenging roles. For example, ellagic acid has demonstrated to attenuate the MDA and reduce SOD activity in gingival tissue of diabetic rat (Al‐Obaidi, Al‐Bayaty, Al Batran, Hussaini, & Khor, 2014). On the other hand, *p*‐coumaric acid was evidenced to decrease the production of MDA which further reduces the risks of atherosclerosis (Guven et al., 2015).

**Table 2:** Lipids parameters, ROS, liver functional enzymes, and blood glucose of experimental rats

		Normal Control	Diabetes Control	PE Treatment
Blood lipid	HDL‐C (mmol/L)	1.45 ± 0.23 b	0.92 ± 0.22 a	1.57 ± 0.07 c
	LDL‐C (mmol/L)	0.37 ± 0.01 a	0.71 ± 0.14 c	0.45 ± 0.09 ab
	TG (mmol/L)	0.80 ± 0.05 a	1.35 ± 0.08 c	1.20 ± 0.02 b
	T‐CHO (mmol/L)	2.48 ± 0.42 a	2.97 ± 0.12 a	2.63 ± 0.12 a
ROS	T‐SOD (U/mL)	0.42 ± 0.02 a	0.54 ± 0.04 ab	0.48 ± 0.05 a
	T‐AOC (U/mL)	2.19 ± 1.44 a	1.65 ± 0.10 a	6.13 ± 0.82 b
	MDA (nmol/mL)	6.74 ± 0.49 a	8.43 ± 0.53 b	6.05 ± 1.19 a
Liver Function	AKP (U/100 ml)	43.81 ± 3.64 a	366.95 ± 10.99 c	320.40 ± 28.16 b
	GOT (U/L)	47.34 ± 2.42 a	64.93 ± 2.35 c	57.36 ± 4.97 b
	GPT (U/L)	37.37 ± 1.23 a	64.07 ± 3.29 c	56.13 ± 3.58 b
Blood Glucose	1W (mmol/L)	5.43 ± 0.50 a	10.42 ± 1.33 c	8.18 ± 0.37 b
	2W (mmol/L)	5.07 ± 0.38 a	10.64 ± 0.96 b	9.20 ± 1.18 b
	3W (mmol/L)	5.16 ± 0.34 a	14.37 ± 1.21 c	11.80 ± 1.10 b
	4W (mmol/L)	5.18 ± 0.47 a	21.27 ± 1.83 c	18.65 ± 1.25 b
	5W (mmol/L)	5.35 ± 0.35 a	24.88 ± 2.16 c	20.06 ± 2.37b
	6W (mmol/L)	6.17 ± 0.56 a	27.81 ± 3.54 b	26.97 ± 1.24 b

Abbreviation: W, Week.

Different letters in the table indicate a significant differences (*p* < 0.05).

The T‐AOC of blood can reflect overall cellular endogenous antioxidative capability (Tong, Lin, Lippi, Nie, & Tian, 2012). Due to the oxidative stress and inflammatory reaction, T‐AOC decreased from 2.19 ± 1.44 U/mL in NC to 1.65 ± 0.10 U/mL in DC (Table 2), while it increased to 6.13 ± 0.82 U/mL (Table 2). It has been reported that phenolics such as ellagic acid, gallic acid, and coumaric acid had strong free radical scavenging activities (Guven et al., 2015). Additionally, other herbs species such as Ilex paraguariensis and panax notoginseng were able to improve plasma T‐AOC activity (Hong et al., 2015; Sánchez Boado, Fretes, & Brumovsky, 2015). Thus, the phenolics in PE could mediate the T‐AOC activity in diabetic rats by inhibition of ROS and amelioration of oxidative stress (Guven et al., 2015).

#### 3.3.2 Blood glucose

Generally, insulin is responsible for mediating glucose uptake (Maria et al., 2015). Once the pancreas function has been suppressed, there will be a resistance of insulin secretion resulting in metabolic syndrome such as hyperglycemia (Maria et al., 2015). Therefore, the increase in blood glucose could be used as a hallmark of diabetes. In the current study, the blood glucose levels of NC, DC, and PE were monitored for 6 weeks. Table 2 shows NC had a relatively stable blood glucose ranging from 5.07 ± 0.38 mmol/L to 6.17 ± 0.56 mmol/L throughout 6 weeks experiment. However, it was approximately twice higher in DC at 1W and four times higher in DC at 6W compared with NC (Table 2) due to destruction of pancreatic insulin‐secreting β‐cells induced by STZ. PE demonstrated the inhibition performance from 1W to 5 W and had the best inhibitory effect at 5W which inhibited about 20% of blood glucose compared with DC (Table 2). At 6W, there was no significant difference between DC and PE (Table 2). The reversal of blood glucose changes with administration of the polygonum cuspidatum extract proved that insulin deficiency had been improved. This might attribute to an increase in glucose utilization through mitochondrial respiratory chain which promotes peripheral glucose utilization by enhancing anaerobic glycolysis (Coller, 2014). In the study of Saibabu et al. (2015), treatment with ellagic acid markedly elevated the insulin‐secreting activity from pancreatic β‐cell, which attenuated the dyslipidemia. On the other hand, ellagic acid could indirectly attenuate hypoglycemic effects through regulating blood glucose homeostasis and glucose uptake which were mediated through AMP‐activated protein kinase (AMPK) activation (Li, Yao, & Li, 2017). It has been addressed that cinnamic acid itself is not able to stimulate glucose uptake; however, it exhibited a synergistic effect with other phenolics on the uptake of glucose (Adisakwattana, 2017). Similarly, *p*‐coumaric acid also induced the glucose uptake and exerts insulin‐like activity in human omental adipocytes as reported in the study of Scazzocchio et al. (2011).

#### 3.3.3 Lipid parameters

Relative insulin deficiency plays a role in creating the features of diabetic. Dyslipidemia is recognized by the elevation of plasma TG and LDL‐C, as well as lower HDL‐C (Taskinen, 2005). In STZ‐induced diabetic rats, increasing levels of LDL‐C and CHO were observed due to the stimulation of hepatic triglyceride synthesis which further over produce TG and LDL‐C (Mohan, Jesuthankaraj, & Ramasamy Thangavelu, 2013). In this study, the effect of PE on lipid parameters, including CHO, TG, HDL‐C, and LDL‐C, in diabetic rats was investigated. As shown in Table 2, DC had significantly twice LDL‐C (0.71 ± 0.14 mmol/L) than NC (0.37 ± 0.01 mmol/L); however, it was only 0.45 ± 0.09 mmol/L in PE. Approximately 12% of TG was inhibited in PE compared with DC which was associated with a decrease of the LDL‐C fraction (Table 2). Compared with DC (2.97 ± 0.12 mmol/L), A slight reduction of CHO was observed in PE (2.63 ± 0.12 mmol/L). PE maintained a similar level of HDL‐C (1.57 ± 0.07 mmol/L) with NC (1.45 ± 0.23 mmol/L) (Table 2). It might be the abundant phenolics, especially ellagic acid, *p*‐coumaric acid, and coumarin attributed to the ameliorative effect of lipids by hepatic LDL receptor site activity (Harnafi et al., 2013). For example, the lipid lowering effect of ellagic acid and *p*‐coumaric acid has been reported to be associated with the decreased alteration in lipoprotein fractions in STZ‐induced diabetic rats (Guven et al., 2015; Malini, Kanchana, & Rajadurai, 2011). Rich in coumarin, the cinnamon was investigated to generate CHO lowering effect (Ranasinghe et al., 2012). Another possible mechanism was described in the study of Das and Barman (2012) on molecular basis. Similar to *Punica granatum* leaves, polygonum cuspidatum extracts with the presence of phenolics could increase LDL‐C receptor mRNA levels, which, in turn, speed up hepatic uptake and degradation of LDL‐C, resulting in a decrease in serum LDL‐C levels. Thus, PE can be absorbed, metabolized, and biologically active in biological system and had the potential of maintaining lipid profiles in diabetic rats.

#### 3.3.4 Liver function

The liver is responsible for the maintenance of blood glucose biological system. Targeting key metabolic and regulatory hepatic enzymes such as GOT, GPT, and ALP are associated with glycolysis and gluconeogenesis (Adisakwattana, 2017). Thus, the control of GOT, GPT, and ALP activities could serve as feasible approaches for diabetes treatment (Han, Kang, Kim, Choi, & Koo, 2016). The hepatotoxic effect of STZ was reflected by the increased activities of serum GOT, GPT, and ALP due to liver dysfunction which led to the leakage of these enzymes from the liver cytosol into the blood stream (Karan, Mondal, Mishra, Pal, & Rout, 2013).

In this study, PE reduced AKP activity from 366.95 ± 10.99 U/100 ml in DC to 320.40 ± 28.16 U/100 ml (Table 2). However, PE had difficulty in generating a reversible effect on AKP in diabetic rats. Due to the leakage of these enzymes from the liver cytosol into the blood stream, an elevation of GOT and GPT activity of plasma was observed in DC (Table 2). The GOT and GPT activities in DC were significantly higher than those in NC (Table 2). However, with the administration of PE, the hepatoprotective effect was observed which normalized GOT and GPT activities to 57.37 ± 4.97 U/L and 56.13 ± 3.58 U/L, respectively (Table 2). The improvement of the liver function‐related enzymes might be attributed to the phenolics in polygonum cuspidatum, especially cinnamic acid. In the report of Rajeshwari et al. (2013), cinnamic acid has been examined to lower the GOT and GPT activities in the cinnamon‐fed diabetic rats. On the other hand, cinnamic acid has demonstrated the modulatory effects on gene and protein expression which were related to cellular signaling transduction of insulin‐targeting organs (Adisakwattana, 2017). Other herbs such as *Pseudarthria viscidaaqueou*s and *Rhus verciflua* extracts showed a protective action in releasing the elevated levels of GOT and GPT in the liver of diabetic rats (Jung et al., 2006; Kuppusamy, Shirwaikar, Sam, & Kaitheri, 2012). It indicated that the bioactive compounds in polygonum cuspidatum extracts could partially reverse the enhanced gluconeogenic activity by revival of insulin secretion to normal levels (Kuppusamy et al., 2012). Besides, significantly reduced activity of ALP was observed in *V. amygdalina‐* and *Psidium guava*‐treated diabetic rats, suggesting hepatoprotective potentials of herbal plants extracts. (Khan, Shanmugavalli, Rajendran, Bai, & Sorimuthu, 2013).

## 4 CONCLUSION

The polygonum cuspidatum extract significantly reduced the AGE formation and protein glycation in a protein sugar‐rich food system. It could also help attenuate the blood glucose level and normalize the serum lipid parameters including LDL‐C, HDL‐C, and TG in diabetic rat. Additionally, it exhibited the antihyperlipidemic, hepatoprotective, and antioxidant function as well. Therefore, the results of this study indicated that polygonum cuspidatum has a potential medication application for treatment of diabetic patients and reduction of unhealthy compounds in food products.

## CONFLICT OF INTEREST

The authors confirm that there are no known conflicts of interest.

## ETHICAL STATEMENTS

This study has not any potential sources of conflict of interest. All animals were housed and cared for in accordance with the Chinese Pharmacological Society Guidelines for Animal Use.

## References

[fsn31029-bib-0001] Adisakwattana, S. (2017). Cinnamic acid and its derivatives: Mechanisms for prevention and management of diabetes and its complications. Nutrients, 9(2), 163 10.3390/nu9020163 PMC5331594 28230764

[fsn31029-bib-0002] Adisakwattana, S. , Sompong, W. , Meeprom, A. , Ngamukote, S. , & Yibchok‐anun, S. (2012). Cinnamic acid and its derivatives inhibit fructose‐mediated protein glycation. International Journal of Molecular Sciences, 13(2), 1778–1789. 10.3390/ijms13021778 22408423 PMC3291992

[fsn31029-bib-0003] Aldini, G. , Vistoli, G. , Stefek, M. , Chondrogianni, N. , Grune, T. , Sereikaite, J. , … Bartosz, G. (2013). Molecular strategies to prevent, inhibit, and degrade advanced glycoxidation and advanced lipoxidation end products. Free Radical Research, 47(Suppl 1), 93–137. 10.3109/10715762.2013.792926 23560617

[fsn31029-bib-0004] Al‐Obaidi, M. M. J. , Al‐Bayaty, F. H. , Al Batran, R. , Hussaini, J. , & Khor, G. H. (2014). Impact of ellagic acid in bone formation after tooth extraction: An experimental study on diabetic rats. The Scientific World Journal, 2014(2014) , 2006–14. 10.1155/2014/908098 PMC4251085 25485304

[fsn31029-bib-0005] Amin, A. , Tuenter, E. , Cos, P. , Maes, L. , Exarchou, V. , Apers, S. , & Pieters, L. (2016). Antiprotozoal and antiglycation activities of sesquiterpene coumarins from ferula narthex exudate. Molecules, 21(10), 1287 10.3390/molecules21101287 PMC6274357 27681714

[fsn31029-bib-0006] Aminjafari, A. , Miroliaei, M. , Angelova, V. T. , Emamzadeh, R. , Djukic, M. M. , Djuric, A. , & Saso, L. (2016). Antioxidant activity and protective role on protein glycation of synthetic aminocoumarins. Electronic Journal of Biotechnology, 24 (Supplement C), 43–48. 10.1016/j.ejbt.2016.08.004

[fsn31029-bib-0007] Ardestani, A. , & Yazdanparast, R. (2007). Cyperus rotundus suppresses AGE formation and protein oxidation in a model of fructose‐mediated protein glycoxidation. International Journal of Biological Macromolecules, 41(5), 572–578. 10.1016/j.ijbiomac.2007.07.014 17765965

[fsn31029-bib-0008] Atawodi, S. E. , Atawodi, J. C. , Pfundstein, B. , Spiegelhalder, B. , Bartsch, H. , & Owen, R. (2011). Assessment of the polyphenol components and in vitro antioxidant properties of syzygium aromaticum (l.). Merr. & Perry. Electronic Journal of Environmental, Agricultural & Food Chemistry, 10(3), 1970–1978.

[fsn31029-bib-0009] Beserra, A. M. , Calegari, P. I. , Souza Mdo, C. , Dos Santos, R. A. , Lima, J. C. , Silva, R. M. , … Martins, D. T. (2011). Gastroprotective and ulcer‐healing mechanisms of ellagic acid in experimental rats. Journal of Agricultural and Food Chemistry, 59(13), 6957–6965. 10.1021/jf2003267 21644797

[fsn31029-bib-0010] Birben, E. , Sahiner, U. M. , Sackesen, C. , Erzurum, S. , & Kalayci, O. (2012). Oxidative stress and antioxidant defense. The World Allergy Organization Journal, 5(1), 9–19. 10.1097/WOX.0b013e3182439613 23268465 PMC3488923

[fsn31029-bib-0011] Bokaeian, M. , Nakhaee, A. , Moodi, B. , Farhangi, A. , & Akbarzadeh, A. (2010). Effects of garlic extract treatment in normal and streptozotocin diabetic rats infected with Candida albicans. Indian Journal of Clinical Biochemistry, 25(2), 182–187. 10.1007/s12291-010-0033-y 23105907 PMC3453106

[fsn31029-bib-0013] Caengprasath, N. , Ngamukote, S. , Makynen, K. , & Adisakwattana, S. (2013). The protective effects of pomelo extract (Citrus grandis L. Osbeck) against fructose‐mediated protein oxidation and glycation. Excli Journal, 12, 491–502. 26966424 PMC4778338

[fsn31029-bib-0014] Chang, J. S. , Liu, H. W. , Wang, K. C. , Chen, M. C. , Chiang, L. C. , Hua, Y. C. , & Lin, C. C. (2005). Ethanol extract of Polygonum cuspidatum inhibits hepatitis B virus in a stable HBV‐producing cell line. Antiviral Research, 66(1), 29–34. 10.1016/j.antiviral.2004.12.006 15781129

[fsn31029-bib-0015] Coller, H. A. (2014). Is cancer a metabolic disease? The American Journal of Pathology, 184(1), 4–17. 24139946 10.1016/j.ajpath.2013.07.035 PMC3873478

[fsn31029-bib-0016] de Melo, L. V. , & Sawaya, A. C. H. F. (2015). UHPLC–MS quantification of coumarin and chlorogenic acid in extracts of the medicinal plants known as guaco (Mikania glomerata and Mikania laevigata). Revista Brasileira De Farmacognosia, 25(2), 105–110. 10.1016/j.bjp.2015.02.005

[fsn31029-bib-0017] Ding, Y. , Wang, L. , Song, J. , & Zhou, S. (2017). Protective effects of ellagic acid against tetrachloride‐induced cirrhosis in mice through the inhibition of reactive oxygen species formation and angiogenesis. Experimental and Therapeutic Medicine, 14(4), 3375–3380. 10.3892/etm.2017.4966 29042921 PMC5639323

[fsn31029-bib-0018] Elosta, A. , Slevin, M. , Rahman, K. , & Ahmed, N. (2017). Aged garlic has more potent antiglycation and antioxidant properties compared to fresh garlic extract in vitro. Scientific Reports, 7, 39613 10.1038/srep39613 28051097 PMC5209668

[fsn31029-bib-0019] Goldin, A. , Beckman, J. A. , Schmidt, A. M. , & Creager, M. A. (2006). Advanced glycation end products: Sparking the development of diabetic vascular injury. Circulation, 114(6), 597–605. 10.1161/CIRCULATIONAHA.106.621854 16894049

[fsn31029-bib-0020] Guven, M. , Aras, A. B. , Akman, T. , Sen, H. M. , Ozkan, A. , Salis, O. , … Cosar, M. (2015). Neuroprotective effect of p‐coumaric acid in rat model of embolic cerebral ischemia. Iranian Journal of Basic Medical Sciences, 18(4), 356–363. 26019798 PMC4439450

[fsn31029-bib-0021] Han, H. S. , Kang, G. , Kim, J. S. , Choi, B. H. , & Koo, S. H. (2016). Regulation of glucose metabolism from a liver‐centric perspective. Experimental & Molecular Medicine, 48(3), e218 10.1038/emm.2015.122 26964834 PMC4892876

[fsn31029-bib-0022] Harnafi, H. , Ramchoun, M. , Tits, M. , Wauters, J.‐N. , Frederich, M. , Angenot, L. , … Amrani, S. (2013). Phenolic acid‐rich extract of sweet basil restores cholesterol and triglycerides metabolism in high fat diet‐fed mice: A comparison with fenofibrate. Biomedicine & Preventive Nutrition, 3(4), 393–397. 10.1016/j.bionut.2013.03.005

[fsn31029-bib-0023] Ho, C. T. , & Wang, M. (2013). Dietary phenolics as reactive carbonyl scavengers: Potential impact on human health and mechanism of action. Journal of Traditional and Complementary Medicine, 3(3), 139–141. 10.4103/2225-4110.114892 24716169 PMC3924986

[fsn31029-bib-0024] Hong, M. , Li, S. , Tan, H. Y. , Wang, N. , Tsao, S. W. , & Feng, Y. (2015). Current status of herbal medicines in chronic liver disease therapy: The biological effects, molecular targets and future prospects. International Journal of Molecular Sciences, 16(12), 28705–28745. 10.3390/ijms161226126 26633388 PMC4691073

[fsn31029-bib-0025] Jariyapamornkoon, N. , Yibchok‐anun, S. , & Adisakwattana, S. (2013). Inhibition of advanced glycation end products by red grape skin extract and its antioxidant activity. BMC Complementary and Alternative Medicine, 13, 171 10.1186/1472-6882-13-171 23849496 PMC3716656

[fsn31029-bib-0026] Jung, C. H. , Zhou, S. , Ding, G. X. , Kim, J. H. , Hong, M. H. , Shin, Y. C. , … Ko, S. G. (2006). Antihyperglycemic activity of herb extracts on streptozotocin‐induced diabetic rats. Bioscience, Biotechnology, and Biochemistry, 70(10), 2556–2559. 10.1271/bbb.60238 17031059

[fsn31029-bib-0027] Karan, S. K. , Mondal, A. , Mishra, S. K. , Pal, D. , & Rout, K. K. (2013). Antidiabetic effect of Streblus asper in streptozotocin‐induced diabetic rats. Pharmaceutical Biology, 51(3), 369–375. 23406357 10.3109/13880209.2012.730531

[fsn31029-bib-0028] Khan, H. B. , Shanmugavalli, R. , Rajendran, D. , Bai, M. R. , & Sorimuthu, S. (2013). Protective effect of Psidium guajava leaf extract on altered carbohydrate metabolism in streptozotocin‐induced diabetic rats. Journal of Dietary Supplements, 10(4), 335–344. 24237189 10.3109/19390211.2013.830677

[fsn31029-bib-0029] Khangholi, S. , Majid, F. A. , Berwary, N. J. , Ahmad, F. , & Aziz, R. B. (2016). The mechanisms of inhibition of advanced glycation end products formation through polyphenols in hyperglycemic condition. Planta Medica, 82(1–2), 32–45. 26550791 10.1055/s-0035-1558086

[fsn31029-bib-0030] Kuo, C. H. , Chen, B. Y. , Liu, Y. C. , Chang, C. M. , Deng, T. S. , Chen, J. H. , & Shieh, C. J. (2013). Optimized ultrasound‐assisted extraction of phenolic compounds from Polygonum cuspidatum. Molecules, 19(1), 67–77. 10.3390/molecules19010067 24362626 PMC6271919

[fsn31029-bib-0031] Kuppusamy, R. , Shirwaikar, A. , Sam, K. G. , & Kaitheri, S. K. (2012). Antidiabetic activity of Pseudarthria viscida aqueous root extract in neonatal streptozotocin‐induced NIDDM rats. Revista Brasileira De Farmacognosia, 22(5), 1079–1084. 10.1590/S0102-695X2012005000105

[fsn31029-bib-0032] Lazurca, M. , Lazurca, D. , Fetea, F. , Ranga, F. , & Socaciu, C. (2012). Evaluation of the phenolic content in the buds of polygonum cuspidatum sieb. et zucc. LAZURCA, 69(2), 273–280.

[fsn31029-bib-0033] Li, H. , Yao, Y. , & Li, L. (2017). Coumarins as potential antidiabetic agents. Journal of Pharmacy and Pharmacology, 69(10), 1253–1264. 10.1111/jphp.12774 28675434

[fsn31029-bib-0034] Liu, W. , Ma, H. , Frost, L. , Yuan, T. , Dain, J. A. , & Seeram, N. P. (2014). Pomegranate phenolics inhibit formation of advanced glycation endproducts by scavenging reactive carbonyl species. Food & Function, 5(11), 2996–3004. 10.1039/C4FO00538D 25233108

[fsn31029-bib-0035] Malini, P. , Kanchana, G. , & Rajadurai, M. (2011). Antiperoxidative and antioxidant effect of ellagic acid on normal and streptozotocin induced diabetes in albino wistar rats. Research Journal of Pharmaceutical, Biological and Chemical Sciences, 2(3), 24–34.

[fsn31029-bib-0036] Maria, Z. , Campolo, A. R. , & Lacombe, V. A. (2015). Diabetes alters the expression and translocation of the insulin‐sensitive glucose transporters 4 and 8 in the atria. PLoS ONE, 10(12), e0146033 10.1371/journal.pone.0146033 26720696 PMC4697822

[fsn31029-bib-0037] Masaki, H. , Okano, Y. , & Sakurai, H. (1999). Generation of active oxygen species from advanced glycation end‐products (AGEs) during ultraviolet light A (UVA) irradiation and a possible mechanism for cell damaging. Biochimica Et Biophysica Acta, 1428(1), 45–56. 10.1016/S0304-4165(99)00056-2 10366759

[fsn31029-bib-0038] Mitri, J. , & Hamdy, O. (2009). Diabetes medications and body weight. Expert Opinion on Drug Safety, 8(5), 573–584. 19538102 10.1517/14740330903081725

[fsn31029-bib-0039] Mohan, Y. , Jesuthankaraj, G. N. , & Ramasamy Thangavelu, N. (2013). Antidiabetic and antioxidant properties of Triticum aestivum in Streptozotocin‐induced diabetic rats. Advances in Pharmacological Sciences, 2013(2013), 716073. 24416041 10.1155/2013/716073 PMC3876669

[fsn31029-bib-0040] Owczarek, A. , Olszewska, M. , & Gudej, J. (2015). Quantitative determination of ellagic acid and gallic acid in Geum Rivale L. and G. Urbanum L. Acta Biologica Cracoviensia S. Botanica, 56(2), 74–78. 10.2478/abcsb-2014-0021

[fsn31029-bib-0041] Peng, W. , Qin, R. , Li, X. , & Zhou, H. (2013). Botany, phytochemistry, pharmacology, and potential application of Polygonum cuspidatum Sieb.et Zucc.: A review. Journal of Ethnopharmacology, 148(3), 729–745. 23707210 10.1016/j.jep.2013.05.007

[fsn31029-bib-0042] Poulsen, M. W. , Hedegaard, R. V. , Andersen, J. M. , de Courten, B. , Bugel, S. , Nielsen, J. , … Dragsted, L. O. (2013). Advanced glycation endproducts in food and their effects on health. Food and Chemical Toxicology, 60, 10–37. 10.1016/j.fct.2013.06.052 23867544

[fsn31029-bib-0043] Priyadarsini, K. I. , Khopde, S. M. , Kumar, S. S. , & Mohan, H. (2002). Free radical studies of ellagic acid, a natural phenolic antioxidant. Journal of Agricultural and Food Chemistry, 50(7), 2200–2206. 10.1021/jf011275g 11902978

[fsn31029-bib-0044] Raghu, G. , Akileshwari, C. , Reddy, V. S. , & Reddy, G. B. (2017). Attenuation of diabetic retinopathy in rats by ellagic acid through inhibition of AGE formation. Journal of Food Science and Technology, 54(8), 2411–2421. 10.1007/s13197-017-2683-8 28740299 PMC5502036

[fsn31029-bib-0045] Rajeshwari, C. U. , Shobha, R. I. , & Andallu, B. (2013). Oxidative stress and antioxidant effects of herbs and spices in diabetes. Annals of Phytomedicine, 2(2), 13–27.

[fsn31029-bib-0046] Ranasinghe, P. , Perera, S. , Gunatilake, M. , Abeywardene, E. , Gunapala, N. , Premakumara, S. , … Katulanda, P. (2012). Effects of Cinnamomum zeylanicum (Ceylon cinnamon) on blood glucose and lipids in a diabetic and healthy rat model. Pharmacognosy Research, 4(2), 73–79. 22518078 10.4103/0974-8490.94719 PMC3326760

[fsn31029-bib-0047] Saibabu, V. , Fatima, Z. , Khan, L. A. , & Hameed, S. (2015). Therapeutic potential of dietary phenolic acids. Advances in Pharmacological Sciences, 2015(2015), 10 10.1155/2015/823539 PMC4579300 26442119

[fsn31029-bib-0048] Sánchez Boado, L. , Fretes, R. M. , & Brumovsky, L. A. (2015). Bioavailability and antioxidant effect of the Ilex Paraguariensis polyphenols. Nutrition & Food Science, 45(2), 326–335. 10.1108/NFS-08-2014-0079

[fsn31029-bib-0049] Scazzocchio, B. , Vari, R. , Filesi, C. , D'Archivio, M. , Santangelo, C. , Giovannini, C. , … Masella, R. (2011). Cyanidin‐3‐O‐beta‐glucoside and protocatechuic acid exert insulin‐like effects by upregulating PPARgamma activity in human omental adipocytes. Diabetes, 60(9), 2234–2244. 21788573 10.2337/db10-1461 PMC3161313

[fsn31029-bib-0050] Shen, Y. , Prinyawiwatkul, W. , Lotrakul, P. , & Xu, Z. (2015). Comparison of phenolic profiles and antioxidant potentials of the leaves and seeds of Thai holy and sweet basils. International Journal of Food Science & Technology, 50(7), 1651–1657. 10.1111/ijfs.12817

[fsn31029-bib-0051] Sheng, Z. , Gu, M. , Hao, W. , Shen, Y. , Zhang, W. , Zheng, L. , … Xu, Z. (2016). Physicochemical changes and glycation reaction in intermediate‐moisture protein‐sugar foods with and without addition of resveratrol during storage. Journal of Agricultural and Food Chemistry, 64(24), 5093–5100. 10.1021/acs.jafc.6b00877 27218138

[fsn31029-bib-0052] Suantawee, T. , Wesarachanon, K. , Anantsuphasak, K. , Daenphetploy, T. , Thien‐Ngern, S. , Thilavech, T. , … Adisakwattana, S. (2015). Protein glycation inhibitory activity and antioxidant capacity of clove extract. Journal of Food Science and Technology, 52(6), 3843–3850. 26028769 10.1007/s13197-014-1452-1 PMC4444878

[fsn31029-bib-0053] Su, D. , Cheng, Y. , Liu, M. , Liu, D. , Cui, H. , Zhang, B. , … Mei, Q. (2013). Comparision of Piceid and Resveratrol in Antioxidation and Antiproliferation Activities In Vitro. PLoS ONE, 8(1), e54505. 23342161 10.1371/journal.pone.0054505 PMC3546968

[fsn31029-bib-0054] Taskinen, M. R. (2005). Type 2 diabetes as a lipid disorder. Current Molecular Medicine, 5(3), 297–308. 15892649 10.2174/1566524053766086

[fsn31029-bib-0055] Teixeira, J. , Gaspar, A. , Garrido, E. M. , Garrido, J. , & Borges, F. (2013). Hydroxycinnamic acid antioxidants: An electrochemical overview. BioMed Research International, 2013, 251754. 23956973 10.1155/2013/251754 PMC3730368

[fsn31029-bib-0056] Tong, T. K. , Lin, H. , Lippi, G. , Nie, J. , & Tian, Y. (2012). Serum Oxidant and Antioxidant Status in Adolescents Undergoing Professional Endurance Sports Training. Oxidative Medicine and Cellular Longevity, 2012, 741239 10.1155/2012/741239 22577491 PMC3345234

[fsn31029-bib-0057] Venugopala, K. N. , Rashmi, V. , & Odhav, B. (2013). Review on natural coumarin lead compounds for their pharmacological activity. BioMed Research International, 2013(2013), 963248 10.1155/2013/963248 23586066 PMC3622347

[fsn31029-bib-0058] Wiczkowski, W. , Szawara‐Nowak, D. , Sawicki, T. , Mitrus, J. , Kasprzykowski, Z. , & Horbowicz, M. (2016). Profile of phenolic acids and antioxidant capacity in organs of common buckwheat sprout. Acta Alimentaria, 45(2), 250–257. 10.1556/066.2016.45.2.12

[fsn31029-bib-0059] Wu, J.‐W. , Hsieh, C.‐L. , Wang, H.‐Y. , & Chen, H.‐Y. (2009). Inhibitory effects of guava (Psidium guajava L.) leaf extracts and its active compounds on the glycation process of protein. Food Chemistry, 113(1), 78–84.

[fsn31029-bib-0060] Younessi, P. , & Yoonessi, A. (2011). Advanced glycation end‐products and their receptor‐mediated roles: Inflammation and oxidative stress. Iranian Journal of Medical Sciences, 36(3), 154–166. 23358382 PMC3556769

[fsn31029-bib-0061] Zhang, H. , Li, C. , Kwok, S. T. , Zhang, Q. W. , & Chan, S. W. (2013). A review of the pharmacological effects of the dried root of polygonum cuspidatum (hu zhang) and its constituents. Evidence‐Based Complementary and Alternative Medicine, 2013(2013), 208349. 24194779 10.1155/2013/208349 PMC3806114

